# Dataset for rapid state of health estimation of lithium batteries using EIS and machine learning: Training and validation

**DOI:** 10.1016/j.dib.2023.109157

**Published:** 2023-04-19

**Authors:** Muhammad Rashid, Mona Faraji-Niri, Jonathan Sansom, Muhammad Sheikh, Dhammika Widanage, James Marco

**Affiliations:** WMG, University of Warwick, Gibbet Hill Road, Coventry, CV4 7AL, UK

**Keywords:** Retired batteries, 2nd life applications, State of health estimation, Battery grading

## Abstract

This article addresses the objective, experimental design and methodology of the tests conducted for battery State of Health (SOH) estimation using an accelerated test method. For this purpose, 25 unused cylindrical cells were aged, by continual electrical cycling using a 0.5C charge and 1C discharge to 5 different SOH breakpoints (80, 85, 90, 95 and 100%). Ageing of the cells to the different SOH values was undertaken at a temperature of 25 °C. A reference performance test (RPT) of C/3 charge-discharge at 25 °C was performed when the cells were new and at each stage of cycling to define the energy capacity reduction due to increased charge-throughput. An electrochemical impedance spectroscopy (EIS) test was performed at 5, 20, 50, 70 and 95% states of charge (SOC) for each cell at temperatures of 15, 25 and 35 °C. The shared data includes the raw data files for the reference test and the measured energy capacity and the measured SOH for each cell. It contains the 360 EIS data files and a file which tabulates the key features of the EIS plot for each test case. The reported data has been used to train a machine-learning model for the rapid estimation of battery SOH discussed in the manuscript co-submitted (MF Niri et al., 2022). The reported data can be used for the creation and validation of battery performance and ageing models to underpin different application studies and the design of control algorithms to be employed in battery management systems (BMS).

**Specifications Table**

**Table:** 

Subject	Energy
Specific subject area	State of Health Estimation of New and Aged Lithium-Ion Batteries
Type of data	Tabulated Graphical Excel files (.xlsx/.xls) Battery Cycler (Digitron) files (.csv) MATLAB files (.mat)
How the data were acquired	**Electrochemical Testing (Ageing, Performance, and Impedance Test)** **Motivation** A set of experiments defined as (1) reference performance test (RPT) and (2) cyclic ageing test **Reference Performance Test (RPT)** In total 25 LIBs were employed for testing. For each SOH condition, 5 cells were tested. An RPT was performed on each LIB when aged through cycling to SOH values of 100% 95%, 90%, 85% and 80%. As part of RPT, the impedance of each LIB was measured using EIS at five different states of charge (SOC): 5%, 20%, 50%, 70%, and 95%. The ambient temperature of the LIBs was set to 15 °C, 25 °C and 35 °C. Providing a combined dataset of 75 separate test conditions (see Table 1). **Cyclic Ageing Test** The cycling ageing was performed at C/2 currnet charge in CC mode then CV mode (C/20) and 1C current discharge.
Data format	Raw Analysed Filtered
Description of data collection	Electrochemical data (e.g., energy capacity) was collected in .csv output file format from the 10A Digotron cycler (MCT10-6-192HD). EIS impedance data was recorded using the IVIUM Multiplexer (MUX-64) and stored in .xlsx file format.
Data source location	Data source location Institution: WMG, University of Warwick City: Coventry Country: United Kingdom GPS coordinates for collected samples/data: 52.38363378953185, −1.5615186436655097
Data accessibility	Repository name: Mendeley Data DOI:10.17632/mn9fb7xdx6.3[1] Direct URL to data: https://data.mendeley.com/datasets/mn9fb7xdx6/3
Related research article	M. Faraji-Niri, M. Rashid, J. Sansom, M. Sheikh, D. Widanage, J. Marco, “Accelerated state of health estimation of second life lithium-ion batteries via electrochemical impedance spectroscopy tests and machine learning techniques”, Journal of Energy Storage, 58 (2023) 106295. (DOI: https://doi.org/10.1016/j.est.2022.106295) [2]

## Value of the Data

• This dataset represents a complete collection of ageing information for commercially available and widely used lithium-ion batteries that include an assessment of how the retained energy capacity of the cell and impedance changes as a function of increased charge-throughput (ageing). The dataset quantities the inter-dependency between SOC and temperature of LIB impedance at different ageing states: from 100%−80% SOH.
• The datasets can be employed by a range of researchers and engineers engaged in battery-related research. Specifically, in the creation of low-order representations of the battery through equivalent circuit models. Such models are widely employed when simulating electric vehicles and other modes of electrified transport, in which the voltage response of the battery is of primary interest.
• This dataset can also be employed in the creation of battery models to underpin the design of control algorithms within the battery management system (BMS) for SOC and SOH estimation or as one element within the thermal management system. And to explore the use of data-driven methods to quantify battery health linked, in part, to accelerated test techniques such as EIS.
• Both academic researchers and industrial engineers would benefit from this data. While similar data does exist in the public domain, often all that is provided are ad-hod datasets for specific SOC or temperature conditions. This data provides a complete dataset to create and validate their models/analysis, in which the source of the data and the experimental methods employed are fully documented and encompass a range of environmental conditions and ageing states.
• This dataset contains a range of test conditions for a commercially available 21,700 format cell with an NMC 811 chemistry. The experimental methodology can be reused to generate comparable datasets for other LIB formats comprising different cathode materials. Further, the models developed from this data will support the experimental evaluation of these cells towards system integration and control system validation.

## 1 Objective

This data is generated to understand the battery degradation with cycling and subsequent variation in SOH. It also contains information about the changes in battery impedance with temperature and SOC at different SOH break points to correlate the battery SOH with impedance rise. The developed dataset can help identify the SOH of an unknown battery using a quick impedance test. This dataset is related to the article published in the Journal of Energy Storage (DOI: https://doi.org/10.1016/j.est.2022.106295) which adds value to this article by providing complete information about the tests and analysis and can support the further development of battery management system (Table 1).

**Table 1:** Summaries of the different test cases comprised in the dataset. There are five SOH conditions were chosen for the test. On each SOH, five different SOC conditions and three different temperature conditions were used to conduct the EIS test, hence a total of 375 EIS tests have been conducted. And the energy capacity test has been conducted at 5 cells at 25 °C for each SOH on conditions as listed.

EIS - Test Parameter	Test Breakpoint Conditions	Total number of Test Cases
SOH (%)	80, 85, 90, 95 and 100%	5
SOC (%)	5, 20, 50, 70 and 95%	5
Ambient Temperature °C	15, 25, 35	3

Total EIS test cases		75

5 cells per RPT stage, the total number of EIS datasets across the complete experimental design space		**375**

Energy Capacity - Test Parameter	Test Breakpoint Conditions	Total number of Test Cases
SOH (%)	80, 85, 90, 95 and 100%	5
Ambient Temperature °C	25	1

Total energy capacity test cases		5

5 cells per RPT stage, the total number of energy capacity datasets across the complete experimental design space		**25**

## 2 Data Description

All the files are stored in the root folder name “DIB_Data”. The output of the reference performance test (RPT), namely the energy capacity data and the EIS data for the different SOH groups can be found in raw form in the folder name “csvfiles”. This folder contains the following sub-folders and files:

1. Capacity_Check
2. EIS_Test

This data has also been converted to the MATLAB format (MAT) and is stored in the folder named “matfiles”. The structure of this folder is the same as that employed in the “csvfiles” folder above. A full description of each folder and files contained is provided below for completeness and to facilitate efficient data access.

1. Capacity_Check

This folder contains the subfolders for the capacity test data for each SOH condition, namely 100–80% SOH. The syntax of the folder names is defined using the format:

“80per_Cells_Capacity_Check_08122021_020cycle” where:

• 80per: defines the SOH condition, e.g., 80%
• *Cells_Capacity_Check:* name of the test
• 08122021: the date the test was performed, e.g., day/month/year
• 020cycle: the number of ageing cycles after which the energy capacity test was conducted. In this case, the energy capacity data was obtained after 20 cycles of ageing.

Contained within each folder are the associated .csv / .mat files. The naming convention for these files follows the format:

Cellnn_100 SOH_Capacity_Check_25 degC_000cycle

Where

• Cellnn: represents a unique identifier for each LIB used in the experimental research, where nn relates to the cell number
• 100SOH: the state of health of the cell (in this case the cell was new)
• *Capacity_Check:* name of the test
• 100SOH: defines the SOH condition of the cell
• 25degC: the ambient temperature of the LIB when the test was undertaken (note that for this dataset, all energy capacity data was measured at 25 °C).

The structure of the raw data files is as follows:

• Rows 1–15: Header information of the test.
• Row 16: Variable names
• Row 17: Unit of the variables
• Row 18-End: Values of the test variables

Definition of each column for Row 16 with units:

• Column 1: Test step [No Unit]
• Column 2: State of the test, i.e., PAU: Pause, DCH: Discharge, CHA: Charge [No unit]
• Column 3: Time for the step [s]
• Column 4: Total program time [s]
• Column 5: Cycle number [No unit]
• Column 6: Cycle level [No unit]
• Column 7: Procedure: Name of the program [No unit]
• Column 8: Cell voltage [V]
• Column 9: Applied current, 0 for no current, +*x*: charge current, -x: discharge current [A]
• Column 10: Accumulative ampere-hours [Ah]
• Column 11: Ampere hours at previous step [AhPrev]
• Column 12: Accumulative Watt-hours [Wh]
• Column 13: Power [Watt]
• Column 14: Cell temperature [ °C]

The headers, column names and units can only be seen in the .csv files and not in the .mat files. However, data in .mat files have the same structure as explained above for the .csv.

**Fig. 1:**
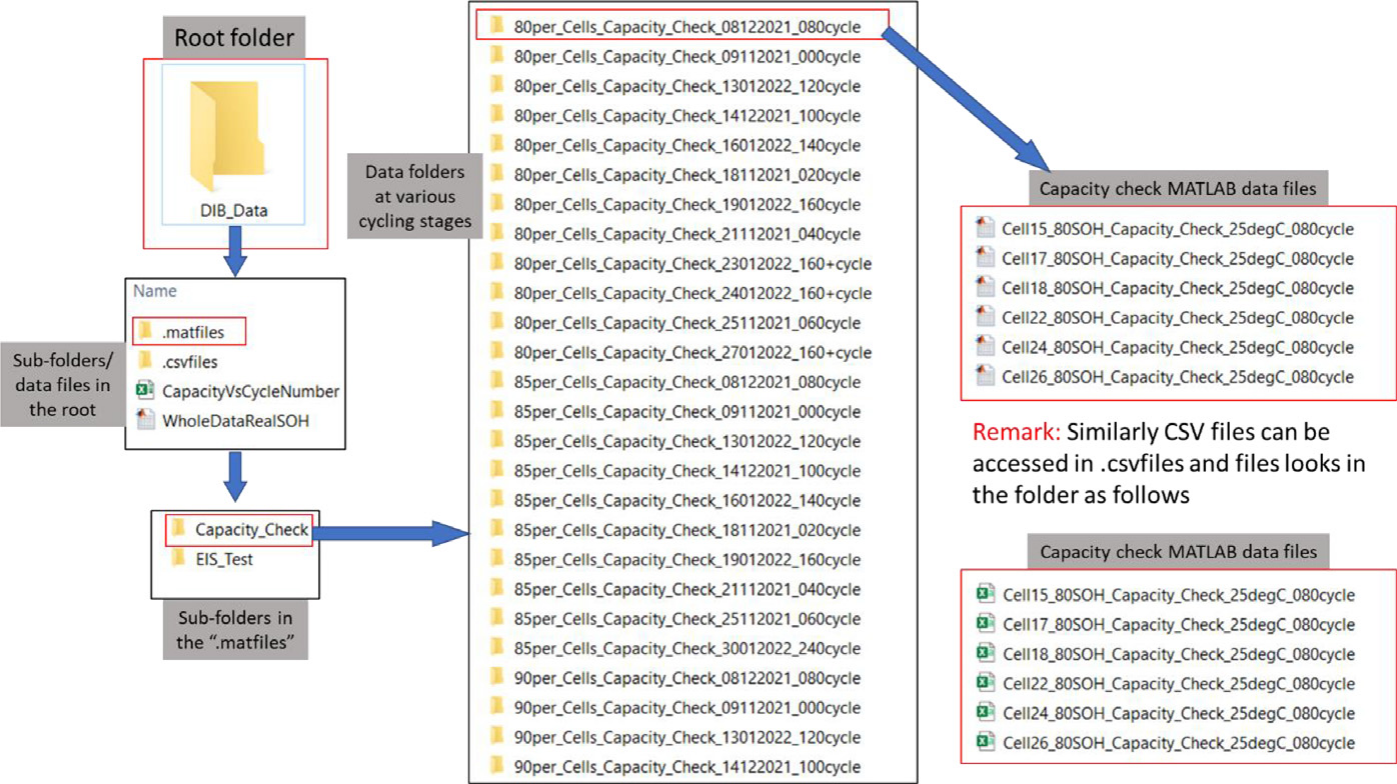
A demonstration to locate capacity check data files in the root folder (DIB_Data). In the root folder, there are two subfolders, .matfiles and .csvfiles. Each of these subfolders contains two folders, Capacity_Cehck and EIS¬_Test. Further going into the Capacity_Check folder, several folders naming “80_per_Cells……” etc. . can be seen. Further to the “80_per_Cells……” folders the capacity check data files “Cell15_80SOH_Capacity_Check ……” can be seen. Example file name “Cell15_80SOH_Capacity_Check_25degC_080cycle” represents Capacity check data for Cell15 at 80% SOH measured at 25degC.

All csv and mat files can be found in the root folder as demonstrated in Fig. 1.

**Fig. 2:**
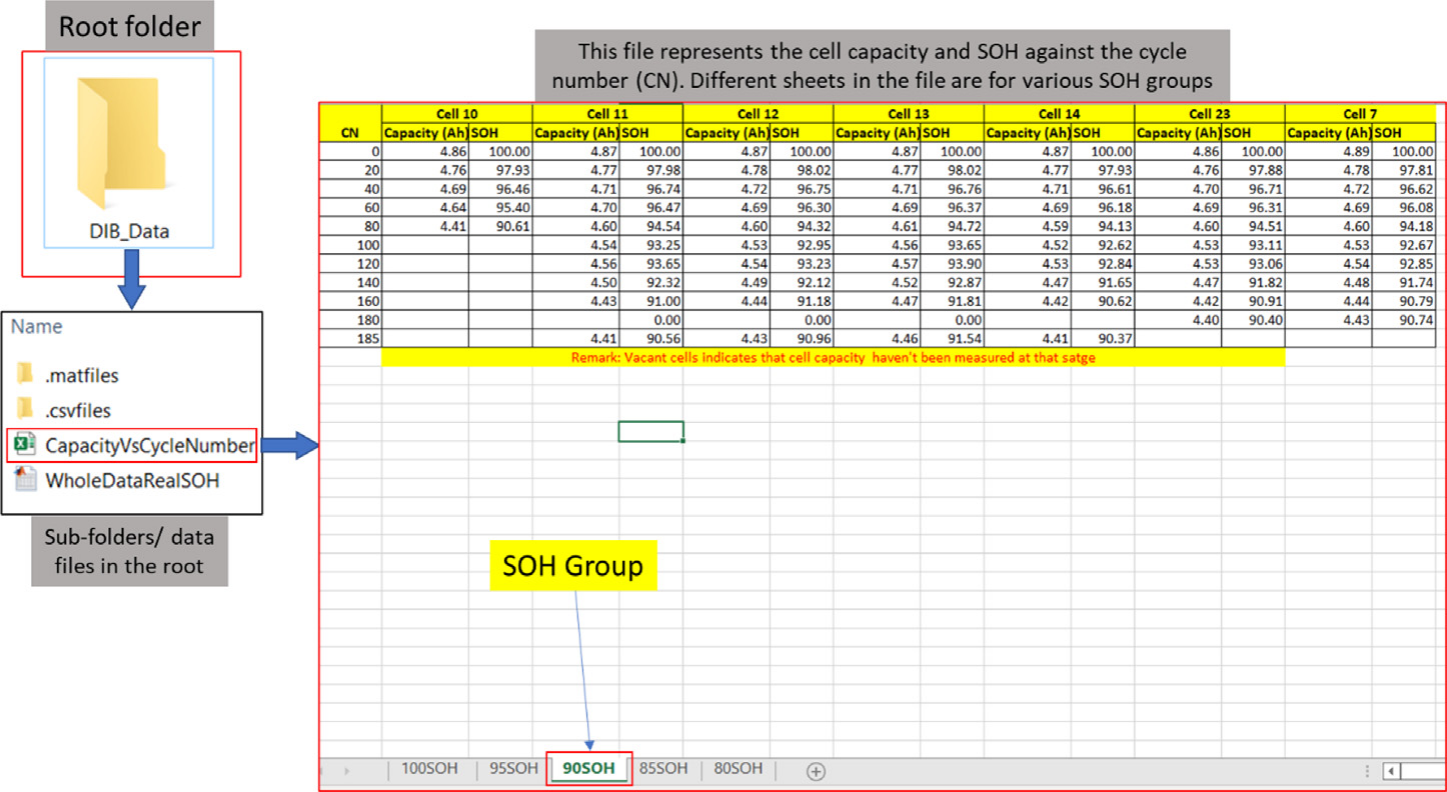
A demonstration to locate capacity vs. cycle number data in the root folder. In the root folder, there is an Excel file “CapacityVsCycleNumber” represents the capacity and SOH of the cells measured at 25degC using the RPT test at various stages of cycling. There are 5 sheets in this Excel file which contain the capacity/SOH data for the cells for different SOH groups.

For completeness, a summary file documents the energy capacity of each cell for different cycle numbers. The file is named: CapacityvsCycleNumber.xlsx. There are five workbooks in this file, and each represents the capacity data for that SOH group of cells. In each sheet, the first column represents the cycle number, and the following set of two columns represents the energy capacity (Ah) and calculated SOH for a cell as shown in Fig. 2. For each cell, three columns of data are defined: cycle number; energy capacity (Ah) and calculated SOH (%). Each workbook in the file represents the data for LIBs from different SOH groups which can also be seen in Fig. 2.

2. EIS_Test

**Fig. 3:**
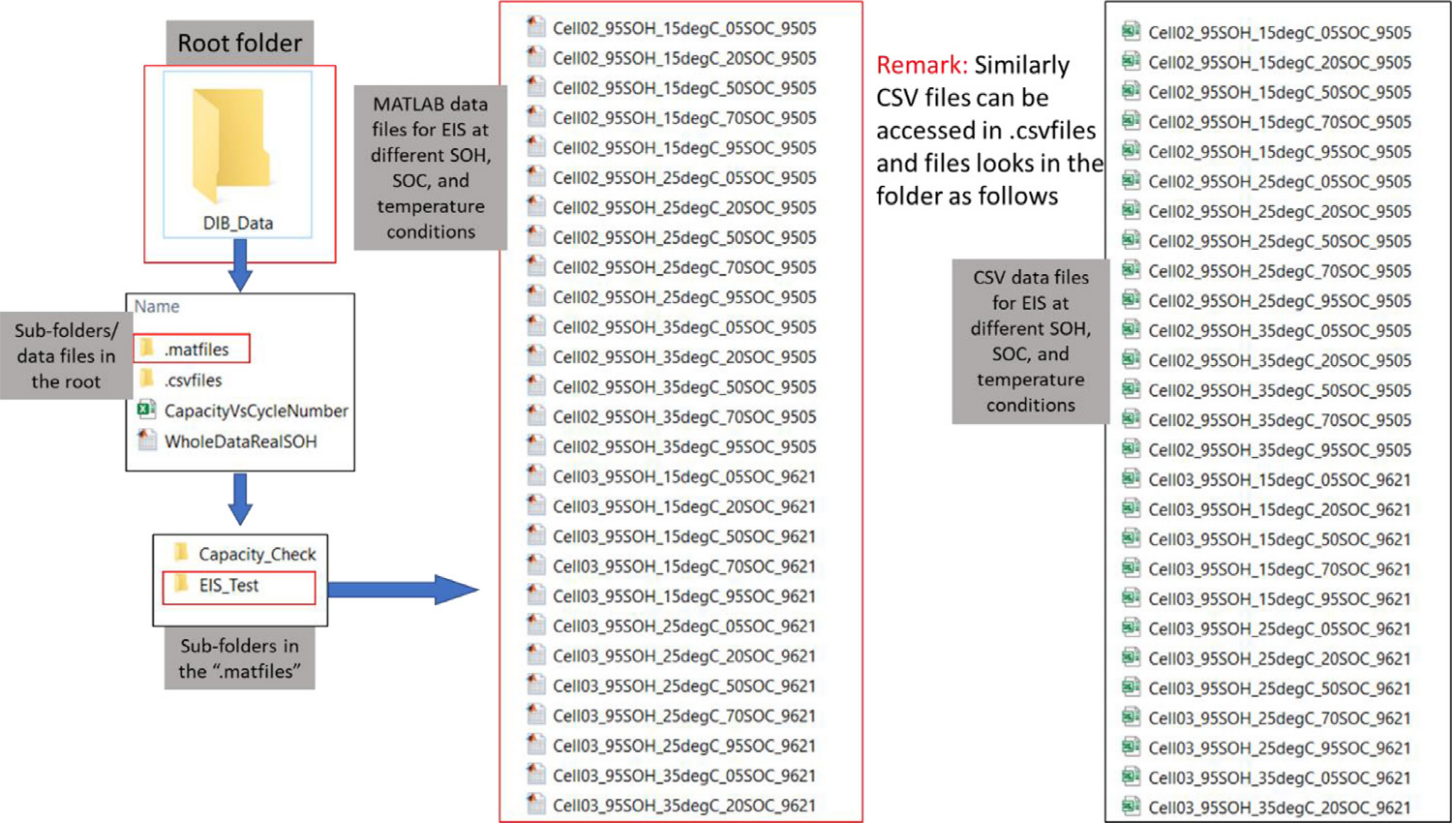
Example to locate EIS_Test data in the root folder. In the root folder, there are two subfolders, .matfiles and .csvfiles. Each of these subfolders contains two folders, Capacity_Cehck and EIS_Test. Further going into the EIS_Test folder, the EIS data files “Cell02_95SOH_15degC……” can be seen. Example file name “Cell02_95SOH_15degC_05SOC_9505″ represents EIS data for Cell2 at 95% SOH measured at 25degC and 5% SOC, the numeric digits in the name represents the actual SOH of the cell multiplied by 100.

This folder contains the data files for all EIS tests conducted across the temperature range: 15, 25 and 35 °C and for the SOC conditions: 5%, 20%, 50%, 70%, and 95%. The naming convention employed for the EIS folder structure is defined below (also demonstrated in Fig. 3):

“Cellnn_100SOH_35degC_95SOC_10,000″

Where

• Cellnn: defines a unique cell identifier, where nn relates to the cell number
• 100SOH: the state of health of the cell when the EIS data was taken (in this case the cell was new)
• 35degC: the ambient temperature for the cell.
• 95SOC: the SOC condition the cell discharged too
• 10,000: represents the actual SOHx100, e.g., 9505 stands for 95.05% of actual SOH

Within the EIS Test folder .xls (.csvfiles/EIS Test) and .mat file (.matfiles/EIS Test) are stored for each associated test condition. Each file is named following the same convention presented above. The structure of both data files is the same:

• Column 1: Frequency (Hz)
• Column 2: Real component of the impedance (Ohms)
• Column 3: Imaginary component of the impedance (Ohms)

**Fig. 4:**
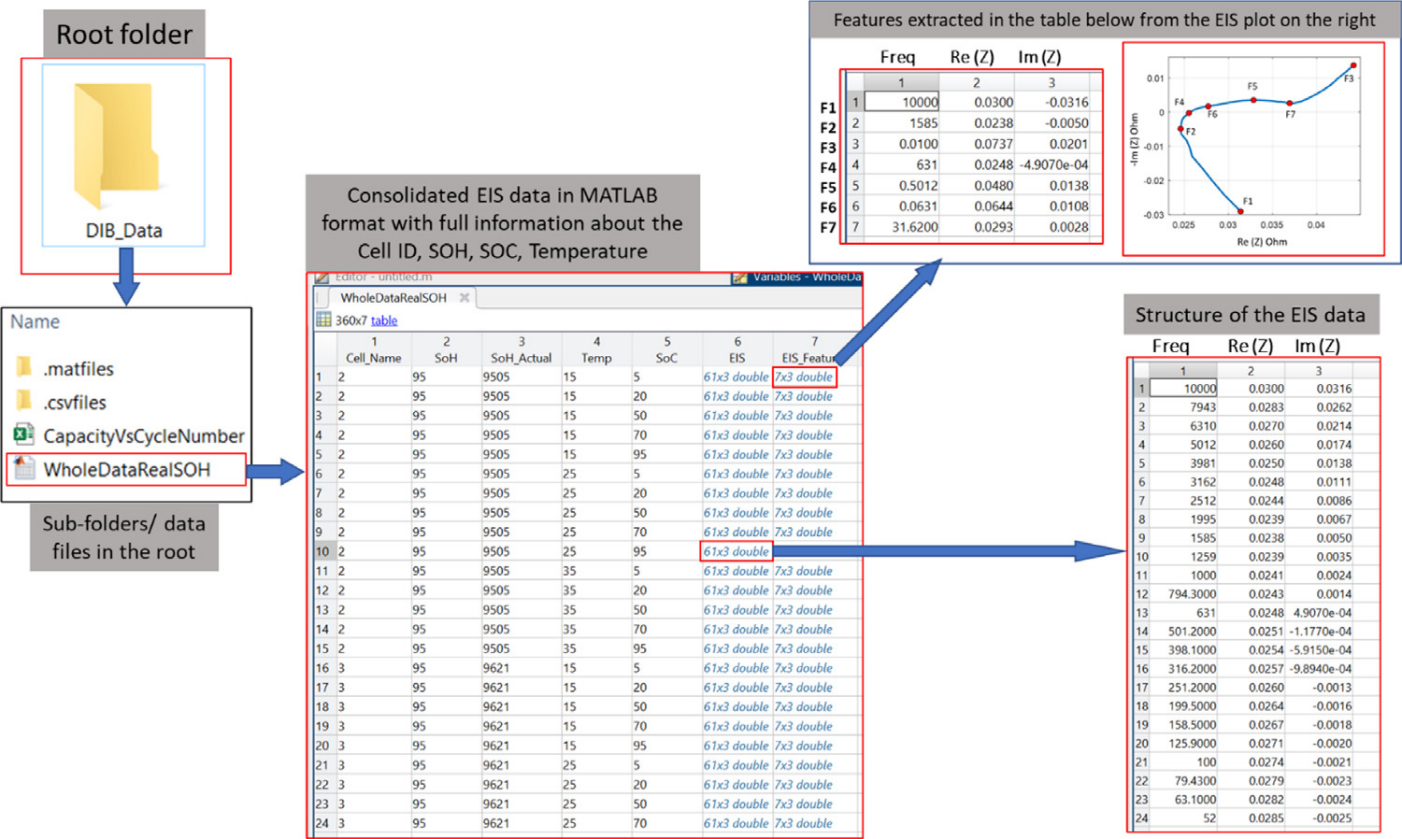
Representation of the whole EIS data with EIS features in the root folder. In the root folder, there is a mat file “WholeDataRealSOH” which contains the full EIS data, EIS features, as well as SOH, temperature, SOC conditions for each cell as named by the columns. Full EIS data is shown in the inset at the right and the EIS features in shown in the inset at the top.

For completeness, a “WholeDataRealSOH.mat” file is provided within the EIS Test folder that summarises the EIS data for each cell (as demonstrated in Fig. 4). The structure of the file comprises a separate column for cell name, SOH, temperature, and SOC. For each measured spectrum, seven features (F1 to F7) from the Nyquist plot are quantified. These were employed extensively when training the machine learning algorithm in the associated research article. A summary of their meaning is provided:

• F1: Highest frequency Datapoint, Freq (Hz), *Re* (Z), Im (Z)
• F2: Point with minimum re value: Freq (Hz), *Re* (Z), Im (Z)
• F3: Lowest frequency datapoint: Freq (Hz), *Re* (Z), Im (Z)
• F4: Zero Crossing Point: Freq (Hz), *Re* (Z), Im (Z)
• F5: local Peak Point: Freq (Hz), *Re* (Z), Im (Z)
• F6: local dip between Zcross and Peak, Freq (Hz), *Re* (Z), Im (Z)
• F7: local dip between peak and end point, Freq (Hz), *Re* (Z), Im (Z)

## 3 Experimental Design, Materials and Methods

Implicit assumptions about battery SOH underpin many studies that evaluate different models for a future battery circular economy. Different end-of-life (EOL) strategies encompassing the recovery and reuse of LIBs are often cited without defining how battery health will be quantified, in the real world, with an accuracy and repeatability that is required to underpin robust decision-making. As a result, stakeholders are often driven to undertake experimental characterisation of LIBs to define key metrics to quantify health and therefore the economic value of the used battery.

The precise battery SOH is widely assessed by a series of charge/discharge cycles which takes several hours to conduct and hence time-consuming and costly [3], [4], [5], [6], [7]. Therefore, a mere experimental approach is insufficient to rapidly grade retired batteries for the optimum energy utilization in potential second-life applications. Hence, a fast-screening method with high accuracy is highly desirable to make the reuse of EV batteries sustainable. So, conducting a set of experiments on the selected batteries and developing a model base on the measured data which can quickly assess the battery SOH will only be a viable solution to this problem. Therefore, in conjunction with the experimental test procedure machine learning method can be a promising tool to assess and validate the battery SOH and the remaining useful life of the batteries for the second life applications. Although, significant effort has been made on using the statistical method and the machine learning method to estimate the battery SOH in view of the 1st/2nd life application [5,[8], [9], [10], [11], [12]. However, these methods are developed based on the data set of very limited operational and environmental conditions such as SOC range and working temperatures. EIS is highly used to assess the battery SOH at a significantly reduced time span [4,[13], [14]. However, the EIS (non-linear) is highly dependent on SOC and temperature and its challenging to attain the right SOC together with the distribution system level impedance to the module and cell level. The model derived based on the limited condition cannot assess the battery SOH in went through the real condition during the first life application, a comprehensive test condition needs to be used to generate the training data. Our research aims to experimentally characterize a set of commercially available LIBs to generate the training and validation dataset for the machine-learning-based SOH prediction algorithm. This dataset helped answer the following questions which have already been discussed and compared in the main article [2]:

a. If the EIS test data can be directly used for the SoH estimation of cells or feature engineering is necessary?
b. What is the configuration of a ML model for SoH estimation?
c. How robust the method is to the measurement noise?
d. How generalisable are the ML-based models to real-world uncertainties arising from variations in test equipment?
e. What is the minimum amount of conditioning information required for an accurate SoH estimation?

As discussed above, the experimental research comprised of (1) an RPT to quantify key cell attributes such as energy capacity and impedance at different states of charge and within different ambient temperatures and (2) cyclic ageing to degrade the LIBs from their initial new condition to 80% SOH. Within the context of this research, SOH is defined as the ratio of the energy capacity measured for the cell at a given cycle number relative to the energy capacity measured when the cell was new.

(1) $$SOH=\;\frac{\text{Current}\;\text{Capacity}}{\text{Reference}\;\text{Capacity}}\times \;100$$

## 4 Experimental Process

### 4.1 Reference Performance Test (RPT)

In total 25 LIBs were employed for testing. For each SOH condition, 5 cells were tested. An RPT was performed on each LIB when aged through electrical cycling to SOH values of 100% 95%, 90%, 85% and 80%.

To measure the retained energy capacity, LIBs were stored in a thermal chamber at 25 °C. They were allowed to soak for one hour to allow them to equilibrate. Each LIB was then charged using a C/3 constant current profile to 4.2 V at which point the LIB was held at this voltage and charged in constant voltage (CV) mode until the value of the charging current reduced to C/20. Once fully charged, the cells were relaxed for one hour to minimize the concentration and potential gradients caused due to the application of the current and to avoid any subsequent effect on the discharge process. LIBs were discharged using a C/3 constant current to a voltage of 2.5 V. The energy extracted during discharge is used to define the cells’ energy capacity.

As part of each RPT, the impedance of each LIB was measured using EIS at five different states of charge (SOC): 5%, 20%, 50%, 70%, and 95%. The ambient temperature of the LIBs was set to 15 °C, 25 °C and 35 °C. Providing a combined dataset of 75 separate test conditions (see Table 1). Thermal stability and homogeneity were provided by immersing the cells in dielectric oil. The temperature of the oil was maintained by an external circulation unit. For each EIS test condition, fully charged LIBs were allowed to rest in the oil for 4 h to eliminate any temperature variation between cells. LIBs were discharged using a C/3 current to the target SOC and allowed to equilibrate for 4 h before the EIS measurement was made. To measure the spectrum, a sinusoidal current of 250 mA, between the frequency range: 10kHz-10mHz was applied to each cell

### 4.2 Cyclic Ageing Test

The cycling ageing was performed by continually charging - discharging the cells at C/2 in CC mode to 4.2 V followed by a CV mode until the charge current was reduced to C/20. During discharge, a 1C discharge current is applied until the cell voltage is reduced to 2.5 V. Cells were conditioned to a temperature of 25 °C during electrical loading through oil immersion, in line with the EIS measurements as part of the RPT.

A summary of the experimental steps is provided below.

**Step 1**: A set of RPT experiments were performed on 25 new LIBs (SOH = 100%). After energy capacity and EIS characterization, five cells were held in storage to support future validation activities.

**Step 2**: 20 LIBs continued to be electrically cycled (following the ageing test profile defined above) until the cells had degraded to a SOH equal to 95%. As highlighted in the associated data files, SOH variations of +/- 1.2% can be observed. This is due to subtle cell-to-cell variations meaning differences in energy capacity and the fact that it is not possible to exactly *synchronize* the ageing of different cells under electrical load. At this point, five cells were selected for RPT characterization. As defined above, this comprised an energy capacity measurement at 25 °C and an impedance measurement for SOC conditions: 5, 20, 50, 70 and 95 and at temperatures: 15 °C, 25 °C and 35 °C. Characterization of 5 cells underpins greater confidence in the efficacy of the dataset and the identification of any cell-to-cell variations that may exist. For all EIS measurements, thermal stability was achieved by immersing the LIBs within dielectric oil set to the desired temperature.

**Steps 3–5** follow the same process defined in Step 2. The set of LIBs was cycled to SOH breakpoints of 90%, 85% and 80%. At each SOH condition, 5 cells were removed for RPT characterisation.

The dataset generated using this approach has been published in the Mendeley repository and can be accessed at ref [1] and using this data set a machine learning model has been developed which can estimate the battery SOH within an error of 1.1% [2].

### 4.3 Lithium-Ion Battery Technology and Equipment

25 unused LGM50 lithium-ion LIBs with a rated energy capacity of 5 Ah were chosen. The cells comprise a nickel-manganese-cobalt (NMC 8111) cathode within a 21,700 cylindrical cell format. The cells have a defined maximum current of 0.7C in charge and 1.5C in discharge.

Direct Current (DC) excitation of the LIBs for charge and discharge employed a 10A Digatron Cycler (Model number: MCT10–6–192HD). Electrochemical impedance spectroscopy (EIS) data was measured using an IVIUM Multiplexer (Model number: MUX-64) Thermal conditioning of the cells employed an ESPEC thermal chamber (Model number: PL3J). Temperate control of the dielectric oil employed a Lauda circulation unit (Model number: RP245E).

## Ethics Statements

The authors confirm that this work meets the ethical requirements of the journal.

## Credit Author Statement

**Muhammad Rashid:** Conceptualization, Methodology, Data Curation and Analysis, Writing – original draft preparation, Experimental Planning and Experimentation; **Muhammad Sheikh:** Resources; **Jonathan Sansom:** Project administration; **Mona Faraji-Niri:** Analysis and Visualization. **Dhammika Widanage:** Software; **James Marco:** Writing – review & editing.

## Declaration of Competing Interest

The authors declare that they have no known competing financial interests or personal relationships that could have appeared to influence the work reported in this paper.

## Data Availability

DIB_Data (Original data) (Mendeley Data).
